# The Utility of Risk Factors to Define Complicated *Staphylococcus aureus* Bacteremia in a Setting With Low Methicillin-Resistant *S. aureus* Prevalence

**DOI:** 10.1093/cid/ciad784

**Published:** 2023-12-29

**Authors:** Thomas W van der Vaart, Jan M Prins, Abraham Goorhuis, Bregtje A Lemkes, Kim C E Sigaloff, Veroniek Spoorenberg, Cornelis Stijnis, Marc J M Bonten, Jan T M van der Meer

**Affiliations:** Julius Center for Health Sciences and Primary Care, University Medical Center Utrecht, Utrecht University, Utrecht, The Netherlands; Department of Internal Medicine, Division of Infectious Diseases, Amsterdam UMC, University of Amsterdam, Amsterdam, The Netherlands; Department of Internal Medicine, Division of Infectious Diseases, Amsterdam UMC, University of Amsterdam, Amsterdam, The Netherlands; Department of Internal Medicine, Division of Infectious Diseases, Amsterdam UMC, University of Amsterdam, Amsterdam, The Netherlands; Department of Internal Medicine, Division of Infectious Diseases, Amsterdam UMC, University of Amsterdam, Amsterdam, The Netherlands; Department of Internal Medicine, Division of Infectious Diseases, Amsterdam UMC, University of Amsterdam, Amsterdam, The Netherlands; Department of Internal Medicine, Division of Infectious Diseases, Amsterdam UMC, University of Amsterdam, Amsterdam, The Netherlands; Department of Internal Medicine, Division of Infectious Diseases, Amsterdam UMC, University of Amsterdam, Amsterdam, The Netherlands; Julius Center for Health Sciences and Primary Care, University Medical Center Utrecht, Utrecht University, Utrecht, The Netherlands; Department of Internal Medicine, Division of Infectious Diseases, Amsterdam UMC, University of Amsterdam, Amsterdam, The Netherlands

**Keywords:** *Staphylococcus aureus* bacteremia, complicated bacteremia, risk factors, treatment

## Abstract

**Introduction:**

Recommended duration of antibiotic treatment of *Staphylococcus aureus* bacteremia (SAB) is frequently based on distinguishing uncomplicated and complicated SAB, and several risk factors at the onset of infection have been proposed to define complicated SAB. Predictive values of risk factors for complicated SAB have not been validated, and consequences of their use on antibiotic prescriptions are unknown.

**Methods:**

In a prospective cohort, patients with SAB were categorized as complicated or uncomplicated through adjudication (reference definition). Associations and predictive values of 9 risk factors were determined, compared with the reference definition, as was accuracy of Infectious Diseases Society of America (IDSA) criteria that include 4 risk factors, and the projected consequences of applying IDSA criteria on antibiotic use.

**Results:**

Among 490 patients, 296 (60%) had complicated SAB. In multivariable analysis, persistent bacteremia (odds ratio [OR], 6.8; 95% confidence interval [CI], 3.9–12.0), community acquisition of SAB (OR, 2.9; 95% CI, 1.9–4.7) and presence of prosthetic material (OR, 2.3; 95% CI, 1.5–3.6) were associated with complicated SAB. Presence of any of the 4 risk factors in the IDSA definition of complicated SAB had a positive predictive value of 70.9% (95% CI, 65.5–75.9) and a negative predictive value of 57.5% (95% CI, 49.1–64.8). Compared with the reference, IDSA criteria yielded 24 (5%) false-negative and 90 (18%) false-positive classifications of complicated SAB. Median duration of antibiotic treatment of these 90 patients was 16 days (interquartile range, 14–19), all with favorable clinical outcome.

**Conclusions:**

Risk factors have low to moderate predictive value to identify complicated SAB and their use may lead to unnecessary prolonged antibiotic use.

## Teaser

Risk factors have low to moderate predictive value to identify complicated SAB. Using risk factors as part of the definition of complicated SAB may lead to misclassification and overtreatment.

The clinical spectrum of *Staphylococcus aureus* bacteremia (SAB) varies widely and ranges from uncomplicated peripheral venous catheter infection to widespread disseminated infection [1–3]. SAB is commonly divided in complicated and uncomplicated SAB to guide clinical management. Guidelines recommend to treat uncomplicated SAB for 2 weeks and complicated SAB for 4 to 6 weeks with antibiotics [4, 5]. A frequently used definition of complicated SAB is the one introduced in 2003 by Fowler and coworkers, in which complicated SAB is defined as SAB complicated by infection-related mortality, metastatic or locally complicated infection, embolic stroke, or recurrent SAB [3]. However, this definition includes outcomes that are unknown at the moment a physician needs to decide whether to treat for 2 weeks or longer. To help identify patients with complicated SAB, the same investigators determined risk factors for complicated SAB: community acquisition of bacteremia, persistent fever, persistently positive blood cultures, and skin findings suggesting acute systemic infection. Although the presence of risk factors for complicated SAB does not equate the actual existence of complicated SAB, presence of these (and other) risk factors is now often used to define complicated SAB, even in the absence of proven metastatic or locally complicated infection [6]. For instance, the Infectious Diseases Society of America (IDSA) guidelines for methicillin-resistant *S. aureus* (MRSA) bacteremia define complicated SAB as presence of locally complicated or metastatic infection, or the presence of 1 of several risk factors (presence of prosthetic material, persistent fever, persistently positive blood cultures, and skin findings suggestive of systemic infection) [4].

However, associations between risk factors and occurrence of complicated SAB are poorly validated, and the investigators reporting these risk factors did not recommend including them as part of the definition [3]. The use of risk factors, therefore, implies the possibility of misclassification, either leading to over- and underdiagnosis of complicated SAB, potentially leading to over- and undertreatment with antibiotics.

In a prospective cohort study of patients with SAB, we retrospectively adjudicated all episodes as either complicated or uncomplicated SAB using a reference definition based on clinical course and outcome as proposed by Fowler et al., and quantified associations and predictive values of risk factors for this outcome. Second, we determined the accuracy of categorizing complicated SAB using the IDSA criteria, compared with the reference definition, and estimated the potential consequences for antibiotic use.

## METHODS

### Design and Study Participants

We performed a multicenter prospective cohort study in 7 hospitals in The Netherlands, including all consecutive adult patients with SAB between July 2017 and September 2019. The cohort and its methodology have been described previously [7]. Patients were followed for up to 90 days after the first day of bacteremia. The institutional review board of the Academic Medical Center Amsterdam approved this study (local protocol number METC2017_094), and written informed consent was obtained from all patients.

The duration of antibiotic treatment was at the discretion of the treating physicians. All hospitals had an active antimicrobial stewardship team, emphasizing follow-up blood cultures after 48 hours, echocardiography, and infectious diseases physician consultation as part of routine care. Diagnostic and treatment decisions were made by the treating physicians without involvement of the study team.

### Definitions

All episodes of SAB were classified as complicated or uncomplicated SAB by an adjudication panel of infectious diseases specialists (A. G., B. L., C. S., J. M., K. S., V. S.) who reviewed case vignettes with detailed clinical courses, diagnostic test results, treatment, and outcome, and assigned for each case presence of infection-related mortality. Using the definition described by Fowler et al., all patients who either had infection-related mortality or a complicated infection present at the time of initial hospitalization (eg, endocarditis, septic arthritis, deep tissue abscesses), or embolic stroke, or relapse of infection within 90 days, were categorized as having complicated SAB [3]. This definition is used throughout the manuscript as the reference classification of complicated SAB, to which other definitions and the risk factors are compared. For efficiency reasons, cases with confirmed metastatic infection (endocarditis, spondylodiscitis, septic arthritis) were considered complicated SAB and not reviewed by the adjudication panel. Each case was independently reviewed by 2 members of the adjudication panel. Discrepancies between panel members were discussed with the study research physician (T. v. d. V.), who provided additional clinical information if required. If no consensus could be reached between the panel members, a third infectious disease specialist was consulted. For describing why a case of SAB was designated as complicated, we used a ranked approach (endocarditis > locally or metastatic complicated disease > relapse > infection-related mortality).

We identified 9 risk factors commonly used to predict complicated SAB (Supplementary Table 1). These include: (1) community acquisition of bacteremia; (2) persistent fever after 72 hours; (3) skin manifestations suggestive of systemic infection; (4) positive follow-up blood cultures after 48 hours; (5) presence of permanently implanted prosthetic material; (6) hemodialysis dependence; (7) an unknown focus of infection at presentation; (8) history of endocarditis, active intravenous drug use, or a heart condition predisposing for endocarditis; and (9) delay in start of effective antimicrobial therapy of 48 hours or more after collection of first positive blood culture [3, 4, 8]. For purposes of analysis, we combined history of endocarditis, active intravenous drug use, and a heart condition predisposing for endocarditis as 1 risk factor because these are all risk factors strongly associated with development of endocarditis.

The IDSA classifies SAB as complicated if any of the following characteristics are present: endocarditis or metastatic infection, presence of permanently implanted prosthetic material, skin findings suggestive of systemic infection, positive follow-up blood cultures after 48 hours, and persistent fever after 72 hours [4]. For clarity, throughout the manuscript, patients who meet the IDSA definition for complicated SAB will be referred to as meeting the IDSA definition.

Site of acquisition (nosocomial, community-acquired, or healthcare associated) was classified as defined by Friedman et al. [9]. Comorbidity was classified using the Charlson comorbidity index [10]. Sepsis and septic shock were defined using the Sepsis-3 guidelines [11]. Persistent bacteremia was defined as a positive blood culture 48 hours after start of effective antimicrobial therapy [12]. Infective endocarditis (IE) was diagnosed by applying the modified Duke criteria [13]. Definite focus of infection was the diagnosis made by the treating physician at hospital discharge. Patients could have multiple infectious foci (eg, endocarditis and spondylodiscitis).

Infection-related mortality was defined as death from direct sequelae of the infection (sepsis, embolic stroke, death during infection control surgery) or persistent signs of infection at the time of death, as described earlier [7].

### Analysis

We determined odds ratios (OR) for risk factors using univariable and multivariable logistic regression. For the multivariable models, only variables with a univariable *P* value > .1 were entered into the model. Positive predictive values of risk factors were determined individually and per set (traditional risk factors: community acquisition, persistent fever, persistent bacteremia, skin findings suggestive of systemic infection; and IDSA risk factors: presence of permanent prosthetic materials, persistent fever, persistent bacteremia, skin findings suggestive of systemic infection). Finally, we quantified agreement between classification according to the reference and the IDSA definition and compared antimicrobial treatment duration for patients with and without complicated SAB. Patients who died from infection-related mortality before completing their full intended treatment were excluded when comparing durations of antimicrobial treatment.

Significance was tested at a 2-sided *P* value of .05, and 95% confidence intervals (CIs) are reported for all inferential statistics. All statistical analysis was done in R version 4.1.2 (R Core Team (2019). R: A language and environment for statistical computing. R Foundation for Statistical Computing, Vienna, Austria).

## RESULTS

### Inclusion

Between August 2017 and September 2019, 636 patients with SAB were screened and 490 (77%) were included in the study. Main reasons for exclusion were patient discharged home before consent was possible (46/146), refusal to provide informed consent (29/146), and incapacitated patients without a legal representative (22/146) [7].

### Demographics and Prevalence of Risk Factors

Demographic and clinical characteristics are shown in Table 1. Risk factors for complicated SAB were very common, with 83% (407/490) of patients having at least 1 of the 9 risk factors under study. The most common risk factors for complicated SAB were presence of permanently implanted prosthetic materials (n = 192, 39%) and community acquisition (n = 166, 34%). Presence of risk factors guided additional diagnostic procedures: fluorodeoxyglucose 18F-positron emission tomography/computed tomography ([18F]FDG-PET/CT) was more often performed in patients with community-acquired SAB, persistent bacteremia, and permanently implanted prosthetic materials (*P* < .05 for all); patients with community-acquired SAB and persistent bacteremia were also more likely to undergo transesophageal echocardiography (TEE) (*P* < .05 for all).

**Table 1.** Demographic and Clinical Characteristics of Patients

	All Patients	Uncomplicated SAB	Complicated SAB
	490	194	296
Demographics and comorbidities			
Sex, male	327 (66.7)	131 (67.5)	196 (66.2)
Age, median [IQR]	68 [57–77]	69 [58–76]	67 [57–77]
Diabetes mellitus	156 (31.8)	65 (33.5)	91 (30.7)
Chronic renal failure	135 (27.6)	61 (31.4)	74 (25.0)
*Hemodialysis*	28 (5.7)	14 (7.2)	14 (4.7)
*Active intravenous drug use*	5 (1.0)	0 (0.0)	5 (1.7)
* Heart condition predisposing for endocarditis* ^a^	150 (30.6)	57 (29.4)	93 (31.4)
Previous endocarditis	13 (2.7)	3 (1.5)	10 (3.4)
Native valve disease	88 (18.0)	38 (19.6)	50 (16.9)
Immunosuppressive medication	86 (17.6)	41 (21.1)	45 (15.2)
Charlson comorbidity index	3 [2–5]	3 [2–6]	3 [1–4]
*Any implanted prosthetic material*^b^	192 (39.2)	58 (29.9)	134 (45.3)
Prosthetic heart valve	40 (8.2)	12 (6.2)	28 (9.5)
Cardiac implantable electronic device	54 (11.0)	17 (8.8)	37 (12.5)
Nonvascular prosthetic materials	94 (19.2)	25 (12.9)	69 (23.3)
Prosthetic joints	64 (13.1)	14 (7.2)	50 (16.9)
Place of acquisition			
*Community-acquired*	166 (33.9)	37 (19.1)	129 (43.6)
Healthcare associated	163 (33.3)	58 (29.9)	105 (35.5)
Hospital acquired	161 (32.9)	99 (51.0)	62 (20.9)
Clinical characteristics			
MRSA	10 (2.0)	5 (2.6)	5 (1.7)
Fever^c^	426 (86.9)	172 (88.7)	254 (85.8)
*Persistent fever* ^d^	54 (16.0)	20 (14.7)	34 (16.8)
Septic shock	52 (10.6)	9 (4.6)	43 (14.5)
qSOFA score, median [IQR]	2 [1, 3]	2 [1, 3]	2 [1, 2]
* Skin manifestations*	47 (9.6)	11 (5.7)	36 (12.2)
* Persistent bacteremia*	140 (28.6)	17 (8.8)	123 (41.6)
*Start of effective antimicrobial treatment >48 h*	3 (0.6)	2 (1.0)	1 (0.3)
*Unknown infection focus at presentation*	99 (20.2)	42 (21.6)	57 (19.3)
Management			
Median duration of treatment^e^	25 (15–46)	16 (14–18)	45 (38–60)
Treated for <16 d^e^	141 (36%)	123 (63%)	18 (9%)
Treated for >27 d^e^	191 (52%)	24 (12%)	167 (84%)

All data are n (%) unless otherwise indicated. Risk factors are in italics.

Abbreviations: IQR, interquartile range; qSOFA, quick sepsis-related organ failure assessment; SAB, *Staphylococcus aureus* bacteremia.

^a^Includes native valve disease, prior endocarditis, prosthetic heart valve, and cardiac implantable electronic device.

^b^Patients could have more than one.

^c^Data missing in 2 patients.

^d^Data missing in 42 patients.

^e^Calculated for 394 patients without infection-related mortality.

### Prevalence of Complicated SAB

According to the reference definition, 296 patients (60%) had complicated SAB resulting from endocarditis (n = 90), other metastatic or locally complicated infection (n = 162), relapse (n = 3), and infection-related mortality (n = 41). Table 2 lists prevalence of complicated SAB per final (discharge) diagnosis.

**Table 2.** Proportions of Complicated SAB per Infectious Focus

Focus	Complicated SAB n (%)	Uncomplicated SAB n (%)
Peripheral IV	23 (28.8)	57 (71.3)
Central venous catheter	18 (38.3)	29 (61.7)
Skin and soft-tissue infection	37 (59.7)	25 (40.3)
Urinary tract infection	10 (27.8)	26 (72.2)
Osteomyelitis	30 (100)	0 (0)
Septic arthritis	58 (100)	0 (0)
Spondylodiscitis	45 (100)	0 (0)
Pneumonia/respiratory tract infection	25 (53.2)	22 (46.8)
Endocarditis	90 (100)	0 (0)
Other focus	53 (85.5)	9 (14.5)
Unknown focus	16 (34)	31 (66)

Numbers add up to more than 490 because patients could have more than 1 infectious focus.

Abbreviations: IV, intravenous; SAB, *Staphylococcus aureus* bacteremia.

### Association Between Risk Factors and Complicated SAB

**Figure 1.**
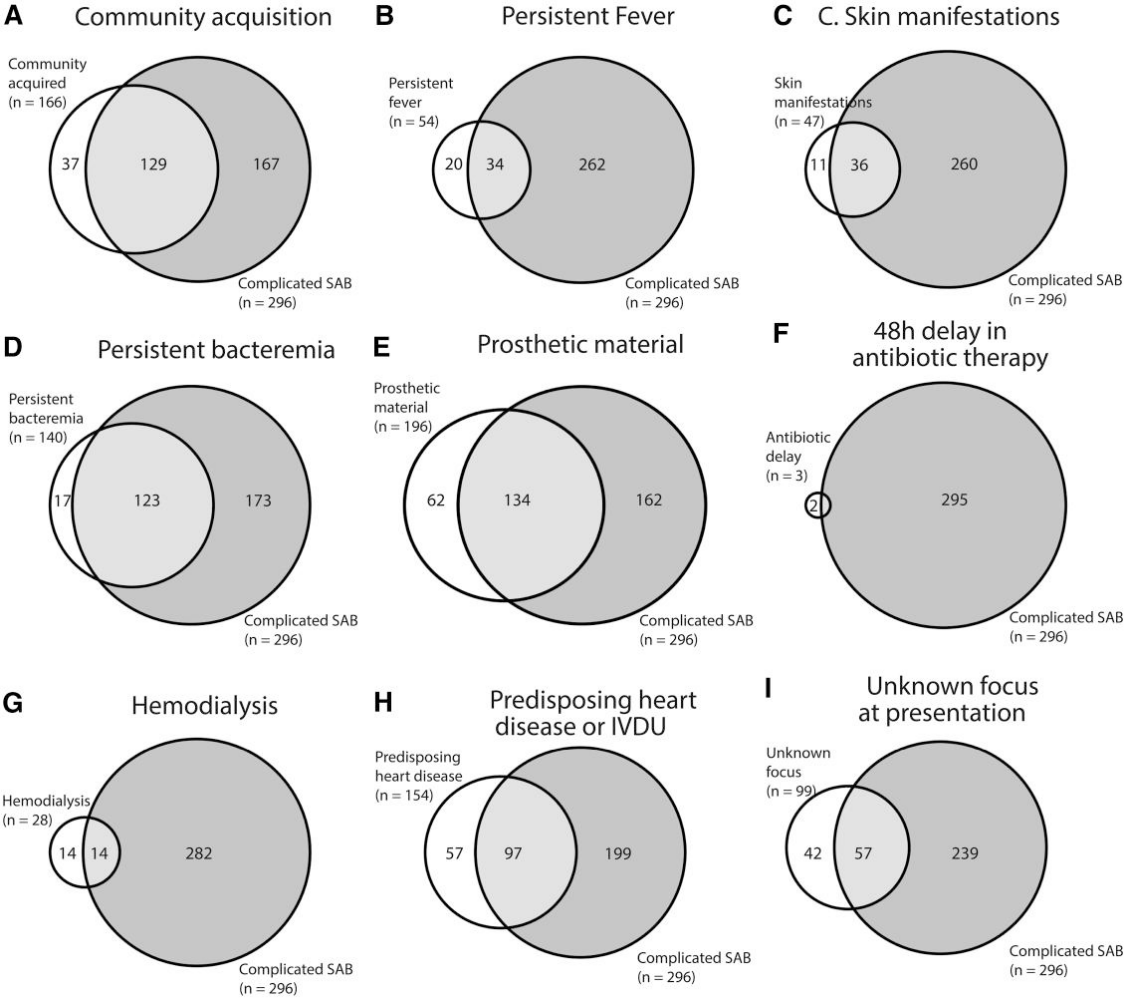
Overlap between risk factors and complicated SAB *A*–*I*, Gray circles represent all patients with complicated SAB; white circles represent all patients with a specific risk factors. The overlap in the circles (shaded light gray) demonstrates the overlap between risk factors and complicated SAB. SAB, *Staphylococcus aureus* bacteremia.

In univariable analyses, of the set of 9 evaluated risk factors, only community acquisition, skin manifestations suggestive of systemic infection, persistent bacteremia, and presence of permanently implanted prosthetic material were associated with complicated SAB (Table 3). In multivariable analysis entering these 4 risk factors, ORs were 6.8 (95% CI, 3.9–12.0) for persistent bacteremia, 2.9 (95% CI, 1.9–4.7) for community acquisition, and 2.3 (95% CI, 1.5–3.6) for presence of prosthetic material (Table 3). Persistent bacteremia had the highest positive predictive value for complicated SAB: 88% (95% CI, 81–93), followed by community acquisition (78%; 95% CI, 71–84), and skin findings suggestive of systemic infection (77%; 95% CI, 62–88). The overlap between risk factors and complicated SAB according to the reference definition is depicted as Venn diagrams in Figure 1. Of note, presence of prosthetic materials was only a risk factor for complicated SAB if the prosthetic device was infected. In a univariable sensitivity analysis that excluded patients with infected prosthetic materials (n = 78), presence of a prosthetic device itself (thus without clinical suspicion of infection) was not predictive of complicated SAB (OR, 0.9; 95% CI, .6–1.4).

**Table 3.** Risk Factors Associated With Complicated SAB

Risk Factor	Univariate OR (95% CI) for Complicated SAB	Multivariate OR (95% CI) for Complicated SAB	Positive Predictive Value (95% CI) for Complicated SAB	Negative Predictive Value (95% CI) for Complicated SAB
Community-acquired	3.3 (2.1–5.0)*	2.9 (1.9–4.7)**	77.7 (70.6–83.8)	48.5 (42.9–54)
Persistent fever at 72 h	1.2 (0.6–2.1)	…	63 (48.7–75.7)	40.8 (35.1–46.8)
Skin manifestations	2.3 (1.1–4.6)*	1.2 (0.5–2.6)	76.6 (62–87.7)	41.3 (36.7–46.1)
Persistent bacteremia	7.4 (4.3–12.8)*	6.8 (3.9–12.0)**	87.9 (81.3–92.8)	50.6 (45.2–55.9)
Any prosthetic material	1.9 (1.3–2.9)*	2.3 (1.5–3.6)**	69.8 (62.8–76.2)	45.6 (39.9–51.5)
Hemodialysis	0.6 (0.3–1.4)	*…*	50 (30.6–69.4)	39 (34.5–43.6)
Predisposing heart condition, previous IE or current IVDU	1.2 (0.8–1.7)	*…*	63 (54.8–70.6)	40.8 (35.5–46.2)
Delay of antimicrobial therapy >48 h after first blood culture	0.3 (0.0–3.6)	*…*	33.3 (0.8–90.6)	39.4 (35.1–43.9)
Unknown focus of infection at presentation	0.9 (0.6–1.4)	*…*	57.6 (47.2–67.5)	38.9 (34–43.9)

Abbreviations: CI, confidence interval; IE, infective endocarditis; IVDU, intravenous drug user; OR, odds ratio; SAB, *Staphylococcus aureus* bacteremia.

^*^Significant at *P* = .1 and added to the multivariate model.

^**^Significant at *P* = .05.

**Figure 2.**
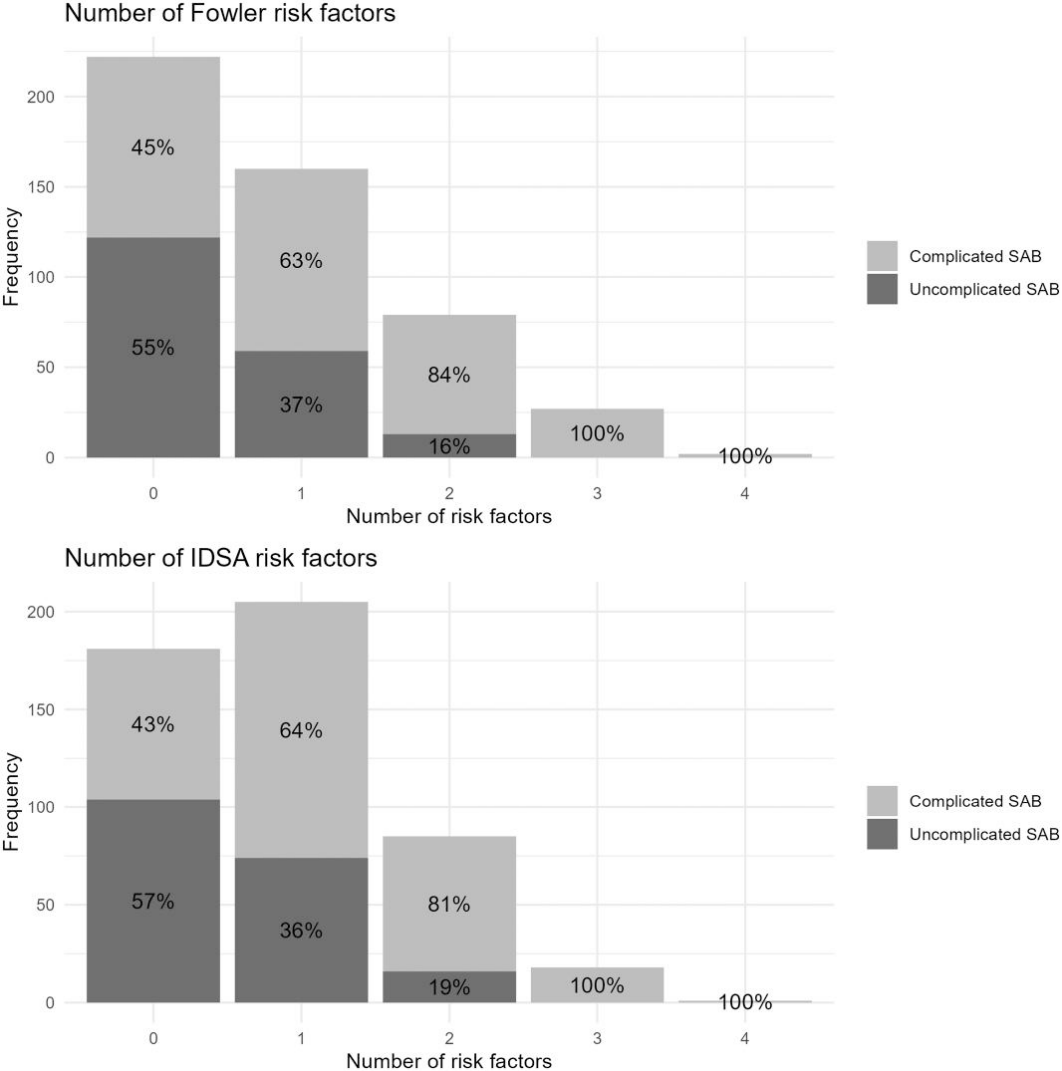
Association between number of risk factors and complicated SAB. Fowler risk factors: community acquisition, persistent bacteremia, skin findings suggestive of systemic infection, and persistent fever. IDSA risk factors: persistent bacteremia, skin findings suggestive of systemic infection, persistent fever, presence of any permanent prosthetic material. IDSA, Infectious Diseases Society of America; SAB, *Staphylococcus aureus* bacteremia.

Both Fowler and coworkers and IDSA have proposed sets of 4 risk factors. Three of the 4 are similar in both sets. This leads to differences in the number of patients with either 0 or 1 risk factor (Figure 2), but this hardly affects the positive predictive values of both sets, being 73.1% (95% CI, 67.4–78.3) and 70.9% (95% CI, 65.5–75.9) for the Fowler and IDSA sets, respectively. Also negative predictive values were comparable (55.0% [48.2–61.6] and 57.5% [49.1–64.8] for the Fowler et al. and IDSA sets, respectively).

### Comparison of IDSA Definition With the Reference Definition

A total of 362 (74%) patients fulfilled the IDSA definition of complicated SAB, of which 272 (75%) were also classified as complicated SAB according to the reference definition (true positives) and 90 (25%) were not (false positives). Most common diagnoses in patients with a false-positive IDSA classification were peripheral phlebitis (n = 23), central line–associated bloodstream infection (n = 14), pneumonia and respiratory tract infections (n = 12), and skin and soft-tissue infections (n = 14). In these 90 false-positive patients, 74 had 1 risk factor and 16 had 2 risk factors. Fifty-eight of 90 patients had permanent prosthetic material, 20 had persistent fever, 17 had persistent bacteremia, and 11 had skin findings suggestive of systemic infection. Twenty-four of 296 patients (8%) did not meet the IDSA definition of complicated SAB but were categorized as such according to the reference definition (false negatives), because of infection-related mortality (n = 22) and relapse infection (n = 2).

### Treatment Duration of Patients With Complicated and Uncomplicated SAB

**Figure 3.**
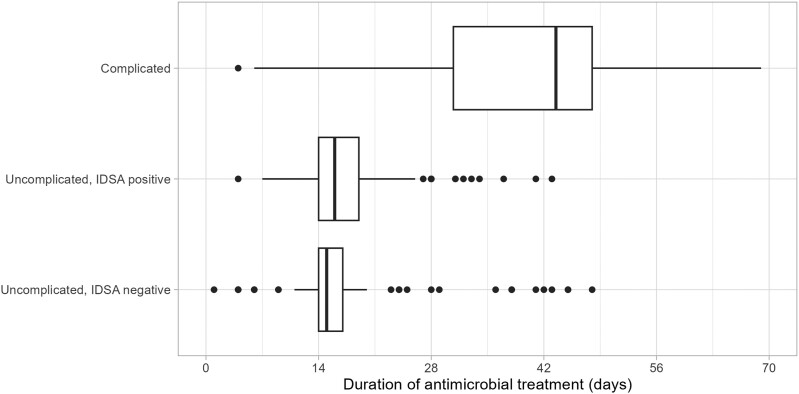
Duration of treatment for complicated and uncomplicated *Staphylococcus aureus* bacteremia.

Median durations of antibiotic treatment were 45 (interquartile range [IQR], 38–60) days in patients with complicated and 16 (IQR, 14–18) days in patients with uncomplicated SAB. In the 90 patients with uncomplicated SAB but with complicated SAB according to the IDSA definition (false positives), median treatment duration was 16 days (IQR, 14–19) days. Because all patients had a favorable clinical outcome, one might conclude that they indeed did not require extended antimicrobial treatment (Figure 3).

In our cohort, 3 patients were classified as having complicated SAB because of relapse infection within 90 days after initial SAB. These patients had been treated with antibiotics for 14, 15, and 19 days, for what was apparently considered uncomplicated SAB by their treating physician. One of these 3 patients would have been classified as complicated by the IDSA definition, and prolonged antibiotic treatment might have prevented recurrent infection. Altogether, application of the IDSA definition in this cohort could have led to unnecessary prolonged antibiotic treatment in 90 patients (18%) and might have prevented 1 episode of recurrent infection.

## DISCUSSION

In this prospective cohort of 490 patients with SAB, 296 (60%) were adjudicated as complicated SAB. Risk factors for complicated SAB are highly prevalent, but both positive and negative predictive values for identifying complicated SAB are suboptimal. Based on IDSA definition criteria, 90 patients with uncomplicated SAB according to the reference would have been classified as complicated SAB and would accordingly have received prolonged antibiotic treatment. Vice versa, 1 episode of recurrent infection might have been prevented with prolonged antibiotic treatment when the IDSA definition had been applied. We conclude that using the IDSA criteria to define complicated SAB is moderately predictive and may increase unnecessary prolonged antibiotic use.

We confirmed the strong association between several risk factors and complicated SAB; notably, persistent bacteremia, community acquisition, and presence of prosthetic material were all independently associated with increased risk of complicated SAB. However, the predictive values of these individual risk factors were too low to reliably diagnose or exclude complicated SAB. For example, 22% of patients with community-acquired bacteremia and 30% of patients with prosthetic material had uncomplicated SAB. The absence of risk factors should not serve as reassurance to clinicians because the majority of patients with SAB will have complicated bacteremia that can be present even without risk factors. Other risk factors commonly thought to be associated with complicated SAB (eg, hemodialysis dependence, unknown focus of infection at presentation) were not associated with complicated SAB in our cohort. This lack of reproducibility may be because risk factors for complicated SAB are often based on a limited number of studies in small or selected populations [14–17].

This combination of insufficient predictive value of confirmed risk factors and potentially overestimated associations of other risk factors enhances the risk of misclassification when using risk factors to classify complicated and uncomplicated SAB. This is illustrated by our hypothetical application of the IDSA definition: 18% of all patients with SAB would have been classified as complicated, whereas they did not have complicated SAB according to the reference definition. The median treatment duration of these patients was 16 (IQR, 14–19) days, with favorable clinical outcome, which supports the statement that these patients indeed did not require prolonged antibiotic treatment. Vice versa, 3 patients treated as uncomplicated SAB by their treating physician had a relapse infection, and 1 of these 3 patients met the IDSA definition of complicated SAB. Accordingly, prolonged antibiotic treatment might have prevented 1 episode of recurrent infection at the cost of 90 patients receiving unnecessary prolonged treatment.

This study was performed in a setting where treating physicians were not instructed to use risk factors alone to diagnose complicated SAB. Dutch guidelines on the treatment of SAB, published after study enrollment was completed, do not provide a definition for complicated SAB, but do recommend treating complicated SAB for 4 to 6 weeks, depending on the final diagnosis [5]. The same guidelines also state that patients with a high risk of a complicated course, but with negative additional diagnostic results, can be classified as having uncomplicated SAB, where 2 weeks of antibiotic treatment would suffice. Indeed, in our cohort, patients with community acquired SAB and persistent bacteremia were more likely to undergo TEE and [18F]FDG-PET/CT than patients without these risk factors, indicating that clinicians used these diagnostic tools to differentiate between uncomplicated and complicated SAB, in search for a definitive diagnosis.

We acknowledge that our results may not immediately lead to a change in practice for many clinicians. Medicolegal risks of deviating from the widely used IDSA definition may prevent physicians from treating for uncomplicated SAB in the presence of a risk factor. Furthermore, physicians may be inclined to treat individual patients longer because the harms of a relapse are perceived as greater than the harms of extending treatment. However, unnecessary extended treatment also exposes patients to the hazards of prolonged antibiotic use and longer hospitalization while also increasing the costs of treatment [4, 18].

Further investigations whether transthoracic echocardiography/TEE and [18F]FDG-PET/CT can be used to safely classify patients as having uncomplicated SAB are warranted. [18F]FDG-PET/CT specifically is an expensive technique with uncertainty about its cost-effectiveness [19, 20]. Biomarkers are another potential tool to help risk stratification, although again their discriminatory ability has not been evaluated in prospective studies [21, 22]. The current definitions of complicated SAB contain a large variety of disease manifestations and this heterogeneity in practice results in incorrect classifications. Alternative algorithms based on treating the underlying diagnosis (eg, endovascular infection, osteoarticular infection, phlebitis) may be a more reasonable approach.

Limitations of our study are the low prevalence of MRSA and intravenous drug use in the cohort, which may decrease generalizability to other settings. The low prevalence of MRSA is of specific importance, MRSA bacteremia requires treatment with different antimicrobials that are traditionally thought to be less effective, and outcomes in MRSA bacteremia tend to be worse [23, 24]. Furthermore, separate risk estimations for prior endocarditis and intravenous drug users were not possible because of their low prevalence in this cohort; we therefore combined them in a composite risk factor “predisposing heart condition, previous IE, or current intravenous drug user.” Last, patient discharge before informed consent was an important reason for noninclusion, and this may have resulted in a higher proportion of complicated SAB.

## CONCLUSIONS

Risk factors have low to moderate predictive value to identify complicated SAB. Using risk factors to define complicated SAB, as recommended by IDSA guidelines, may increase the proportion of patients with complicated SAB by 20%. Because classification is used to guide the duration of antibiotic treatment, this will inadvertently lead to unnecessary prolonged antibiotic use. These findings illustrate that in patients with SAB, better criteria, in addition to reliable additional diagnostic procedures, are needed to optimize clinical management.

## Supplementary Data

Supplementary materials are available at *Clinical Infectious Diseases* online. Consisting of data provided by the authors to benefit the reader, the posted materials are not copyedited and are the sole responsibility of the authors, so questions or comments should be addressed to the corresponding author.

## Supplementary Material

ciad784_Supplementary_Data

## Notes

***Financial support*.** This work was funded by the Amsterdam University Medical Centers and the Universitair Medisch Centrum Utrecht.
