# Supplementary material for: The Utility of Risk Factors to Define Complicated Staphylococcus aureus Bacteremia in a Setting With Low Methicillin-Resistant S. aureus Prevalence
Source: Clin Infect Dis. 2023 Dec 29;78(4):846–54. doi: 10.1093/cid/ciad784 (PMC11006106; doi:10.1093/cid/ciad784)
Supplement: ciad784_Supplementary_Data [file ciad784_supplementary_data.docx]

**SUPPLEMENTAL MATERIALS**

**Supplemental table 1**

| **Risk factor** | **Review/guideline citing this risk factor for complicated SAB** |
| --- | --- |
| Community acquisition | (4, 5, 8, 23, 25) |
| Persistent fever at 72h | (5, 8, 23, 25, 26) |
| Skin manifestations suggestive of systemic infection | (5, 8, 23, 25, 26) |
| Positive follow-up blood culture at 48h | (5, 8, 23, 25, 26) |
| Permanently implanted prosthetic material | (5, 8, 25, 26) |
| Hemodialysis dependence | (5, 23) |
| History of endocarditis | (8) |
| Current IV-drug use | (5, 8) |
| Heart condition predisposing for endocarditis | (5, 8) |
| Delay in start of antimicrobial therapy of >48h after collection of first positive blood culture | (5, 8) |
| Unknown focus of infection at presentation | (5, 8) |

**Additional references to supplemental materials**

25. South Australian expert Advisory Group on Antimicrobial Resistance (SAAGAR). Staphylococcus aureus Bacteraemia (SAB) Management Clinical Guide (Adult). Australian Commission on Safety and Quality in Health Care; 2023. Only only. Availavle from <https://www.sahealth.sa.gov.au/>

26. Brown NM, Goodman AL, Horner C, Jenkins A, Brown EM. Treatment of methicillin-resistant Staphylococcus aureus (MRSA): updated guidelines from the UK. JAC Antimicrob Resist. 2021;3(1):dlaa114.
